# Effectiveness and Adverse Events of Gabapentinoids as Analgesics for Patients with Burn Injuries: A Systematic Review with Meta-Analysis and Trial Sequential Analysis

**DOI:** 10.3390/jcm12155042

**Published:** 2023-07-31

**Authors:** Liang-Jui Chiang, Pei-Chun Lai, Yen-Ta Huang

**Affiliations:** 1 Department of Surgery, National Cheng Kung University Hospital, College of Medicine, National Cheng Kung University, Tainan 704302, Taiwan; 2 Education Centre, National Cheng Kung University Hospital, College of Medicine, National Cheng Kung University, Tainan 704302, Taiwan

**Keywords:** burn, gabapentinoids, gabapentin, meta-analysis, pain, pregabalin, trial sequential analysis

## Abstract

(1) Background: Pain after a burn injury is difficult to endure, and emerging studies aim to ascertain the effects of gabapentin and pregabalin as non-opioid treatment options. (2) Methods: We searched for randomised controlled trials (RCTs) in six databases. The risk of bias was assessed using the RoB 2.0 tool. We performed meta-analysis and trial sequential analysis and used the Grading of Recommendations, Assessment, Development and Evaluation (GRADE) methodology for judging the certainty of evidence (CoE). (3) Results: Five RCTs were included. Compared with placebo, gabapentinoids significantly decreased the pain intensity within 24 h (mean difference (MD) = −1.06, 95% confidence interval (CI): −1.47–−0.65) and from 72 h to 9 days (MD = −0.82, 95% CI: −1.16–−0.48), but not after 3 weeks (MD = −0.44, 95% CI: −1.31–0.42). Opioid consumption (mg/day) was reduced within 24 h (MD = −13.34, 95% CI: −22.16–−4.52) and from 72 h to 9 days (MD = −7.87, 95% CI: −14.82–−0.91). Increased risks of drowsiness (risk ratio (RR) = 3.255, 95% CI: 1.135–9.335) and dizziness (RR = 3.034, 95% CI: 1.006–9.147) were observed, but sensitivity analysis using the Bayesian method showed no increased risk. All endpoints were judged as low to very low CoE. (4) Conclusions: Gabapentinoids offer modest analgesic benefits as a component of multimodal pain management for burn injuries of less than 3 weeks. The adverse effects should be carefully monitored. Large-scale RCTs are warranted for the reinforcement of CoE in clinical use.

## 1. Introduction

Burn-induced pain is noxious and often persists for a long time after the initial injury, placing a burden on the patient and the healthcare system [1,2]. The proposed mechanism of burn pain involves the central and peripheral nervous systems with mixed features of nociceptive, inflammatory and neuropathic pain [1]. The selection of analgesics for burn-induced pain is often multi-modal. Opioids remain the mainstay of pharmacological treatment for burn pain, but their use should be tailored to avoid tolerance, opioid-induced hyperalgesia and overdose [2,3,4]. Pregabalin and gabapentin are γ-aminobutyric acid analogues that bind to the α2δ protein, which inhibits calcium influx and release of excitatory neurotransmitters [5]. The antinociceptive and anxiolytic effects of these gabapentinoids have been utilised in different peripheral neuropathic pain syndromes, but their role in post-burn pain management is still under debate [6,7]. Therefore, we conducted this systematic review with meta-analysis and trial sequential analysis (TSA) to provide additional insights into the role of gabapentinoids in treating burn injuries.

## 2. Materials and Methods

### 2.1. Protocol and Registration

We performed a systematic review in accordance with the latest guidelines of the Preferred Reporting Items for Systematic Reviews and Meta-Analyses statement (PRISMA 2020) [8]. We registered our protocols with updated modification on INPLASY under registration number INPLASY202310007 (doi: 10.37766/inplasy2023.1.0007).

### 2.2. Search Strategy

Two authors (LJ Chiang and PC Lai) performed a systematic search without language restrictions on PubMed, Embase, Scopus, Cochrane Central Register of Controlled Trials (CENTRAL), Cumulative Index to Nursing and Allied Health Literature (CINAHL), China National Knowledge Infrastructure (CNKI) and Google Scholar for randomised controlled trials (RCTs) that compared gabapentinoids with a control in post-burn patients from inception up to 30 December 2022. In addition, we also searched ClinicalTrials.gov and the European Union Drug Regulating Authorities Clinical Trials Database for any ongoing or unpublished trials. We used hierarchical search terms (e.g., Medical Subject Headings) and text word terms to search for articles about ‘burn pain’, ‘post-burn’, ‘pain’, ‘gabapentin’, ‘pregabalin’, ‘mirogabalin’ and ‘analgesics’. The detailed search strategy is shown in Supplementary Table S1.

### 2.3. Selection Criteria

The inclusion and exclusion criteria were determined before the systematic search. After automatic removal of duplicates, the remaining records and reports were screened for eligibility. We included only RCTs based on the following criteria:

(i) Population: patients with burn wounds;
(ii) Intervention: gabapentinoids (gabapentin, pregabalin or mirogabalin);
(iii) Comparison: control group regimen;
(iv) Outcomes: (a) pain score, (b) opioid consumption and (c) adverse effects.

Studies were excluded if they met one or more of the following criteria: (1) article type is review article, case report, case series, retrospective data analysis or non-randomised prospective study; (2) no available or relevant data for meta-analysis; (3) trial comparing any other analgesics in the control group instead of gabapentinoids; (4) pharmacological or non-thermal pain model; and (5) duplicate publication. Any discrepancy was resolved through group consensus. The references of the included studies were cross-checked. LJ Chiang and PC Lai independently reviewed the title, abstract and full text of the studies. Disagreements were resolved by a third reviewer (YT Huang).

### 2.4. Data Extraction

A data collection form was specifically developed for this review, and two authors (LJ Chiang and PC Lai) independently evaluated the full manuscripts of all included trials and performed data extraction. Data extracted from the trials included demographics, drug administration, sample size, number of patients in treatment groups, follow-up period, pain scores, opioid consumption and adverse events. We extracted values from graphs for unavailable numerical data.

The primary outcomes were pain scores and opioid consumption up to 3 weeks after the burn injury. Secondary outcomes were adverse events after administration of gabapentinoids compared with the control group. Any pain score and daily opioid consumption within 3 weeks in the enrolled studies were extracted. We further subdivided them into three groups: within 24 h, from 72 h to 9 days and after 3 weeks. Adverse events of gabapentinoids were calculated, including dizziness, drowsiness, nausea, diarrhoea, constipation, urinary retention and pruritus.

Visual analogue scale (VAS) or numeric rating scores for pain reported as 0–100 were converted into a 0–10 scale for analysis (0, no pain; 10, worst possible pain). Opioid consumption was converted according to parenteral morphine equivalents (MEs). Data presented as median and interquartile range (IQR) were converted into mean and standard deviation (SD) on the basis of the Cochrane Handbook 7.7.3.5 by using mean = median and SD = IQR/1.35 [9].

### 2.5. Statistical Analysis

Dichotomous and continuous outcomes were presented as a risk ratio (RR) and mean difference (MD), respectively, with 95% confidence intervals (CIs). Statistical analysis was performed using Review Manager (RevMan) version 5.4.1 (The Cochrane Collaboration, London, United Kingdom). We utilised the random-effects model for continuous data and the inverse variance heterogeneity (IVhet) model for dichotomous data by using the Microsoft Excel v365 (Microsoft, Redmont, WA, USA) add-in MetaXL 5.3 (EpiGear International, Sunrise Beach, Australia) to calculate pooled estimates of adverse events. We opted for the IVhet model as it is considered a more effective alternative to the traditional random-effects model. Heterogeneities among studies were assessed using I square (I^2^) statistics. An I^2^ value higher than 50% represents substantial heterogeneity. For each outcome, we performed further subgroup analysis according to the different analgesics using the Q-test. For analyses involving three or more randomized controlled trials (RCTs), we assessed publication bias using the Doi plot and the Luis Furuya-Kanamori asymmetry (LFK) index for each endpoint. These methods were employed to effectively detect and visualize the presence of publication bias [10]. An LFK index out of ±1 is defined as asymmetry of the Doi plot and indicated the presence of publication bias. For the sensitivity analysis of zero adverse events, we utilised the random-effects model of the Bayesian approach using the interactive web-based tool MetaInsight (https://crsu.shinyapps.io/metainsightc, accessed on 28 February 2023) and we obtained a 95% credible interval (CrI) [11,12].

TSA was conducted using TSA version 0.9.5.10 beta (Copenhagen Trial Unit, Centre for Clinical Intervention Research, Rigshospitalet, Copenhagen, Denmark) for more than two RCTs in primary endpoints to avoid the risk of spurious results when too few studies and participants were enrolled [13]. Type I and power were set at 5 and 80%, respectively. The O’Brien–Fleming monitoring boundaries using the random-effects model of the Biggerstaff–Tweedie method were applied for hypothesis testing. The mean difference and variance were set as empirical for calculation of required information size (RIS) in continuous data. Model variance-based correction of heterogeneity was chosen.

### 2.6. Quality Assessment and Certainty of Evidence

The risk of bias (RoB) was independently assessed by two authors (LJ Chiang and PC Lai) using the risk-of-bias tool 2.0 (RoB2) [14]. The results were drawn using the “Risk-of-Bias Visualisation tool” [15]. The certainty of evidence (CoE) was assessed by two authors (LJ Chiang and YT Huang) using the Grading of Recommendations, Assessment, Development and Evaluation (GRADE) methodology [16]. CoE can be rated down in any of the five domains: risk of bias, inconsistency, indirectness, imprecision or publication bias. Grading was conducted using the GRADEpro Guideline Development Tool (McMaster University and Evidence Prime, 2022; available from gradepro.org, accessed on 6 May 2023).

## 3. Results

### 3.1. Literature Search

**Figure 1:**
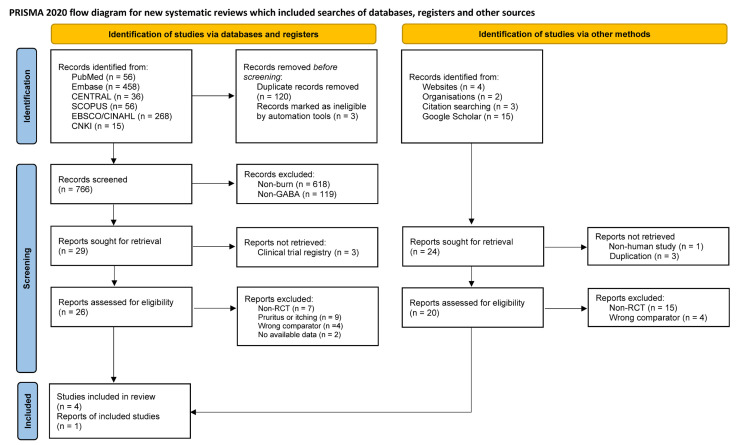
Flow diagram of the preferred reporting items for systematic reviews and meta-analysis (PRISMA) 2020.

The initial literature search retrieved 889 studies from the databases (PubMed, 56; Embase, 458; Cochrane library, 36; SCOPUS, 56; CINAHL, 268; CNKI, 15) and 24 studies via other methods (Figure 1). After removing duplicate and ineligible records through automation tools (EndNote version X9, Clarivate Analytics, Philadelphia, PA, USA), the 766 remaining articles were screened for titles and abstracts. For potentially relevant articles, the full texts were retrieved to determine their eligibility for final analysis. A total of seven studies were excluded due to non-randomised trials, nine studies with unavailable outcomes for pooled estimates, four studies due to different comparators and two studies due to unavailability of data. Finally, five studies were included in the meta-analysis. The results were demonstrated as a PRISMA flowchart according to the PRISMA 2020 statement [8].

### 3.2. Study Characteristics

The characteristics of the included studies are shown in Table 1. All five trials included in this meta-analysis were written in English [17,18,19,20,21]. The source of funding was mentioned in all studies and came from the pharmaceutical industry in two trials [17,18]. All participants of the included trials were adults who experienced a burn injury and were admitted for wound care and possible surgical intervention. In the included trials, three trials (156 participants) evaluated gabapentin and two trials (141 participants) evaluated pregabalin. All the included trials were parallel-designed RCTs and reported baseline analgesics for burn pain. Gabapentinoids were administered as multiple oral doses in four trials [17,18,20,21] (247 participants) and as a single oral dose in one trial [19] (50 participants). Pain scores and opioid consumption were reported in all the included trials. However, the cumulative opioid consumption in the study by Wibbenmeyer et al. did not clarify the time interval, and it was therefore excluded from our analysis [21]. Adverse events were recorded in four trials (207 participants) in the form of text or tables [18,19,20,21]. The analgesic effects and adverse events were followed up for more than one week in two trials (141 participants) [17,18].

### 3.3. Risk of Bias Assessment

**Figure 2:**
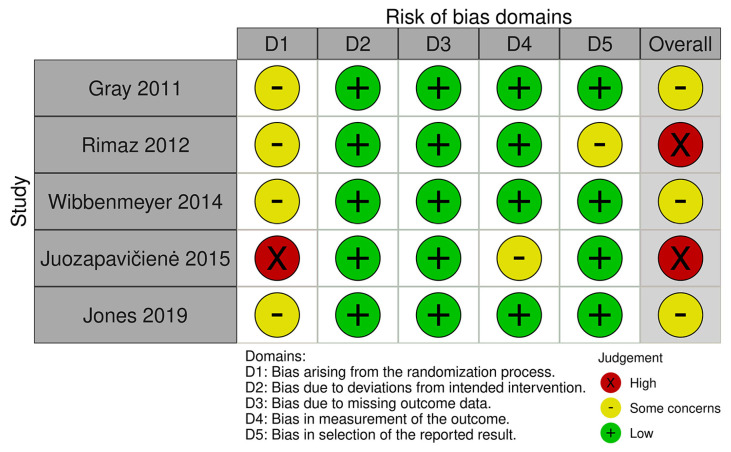
Summary of risk of bias assessment [17,18,19,20,21].

The overall RoB of the five enrolled studies were judged as ‘some concerns’ in three trials and ‘high’ in two trials (Figure 2). In the domain of randomisation, all included studies did not clearly describe the concealment process. Moreover, baseline imbalance was mentioned in the study of Juozapavičienė et al. due to a statistically significant gender difference that might lead to bias. Therefore, the judgment of bias arising from the randomisation process was high in the study of Juozapavičienė et al. and rated as ‘some concerns’ in the four remaining studies. For the domain of measurement of outcome, we judged the study of Juozapavičienė et al. with ‘some concern’ due to lack of blinding with subjected outcomes reported. In the domain of selective reporting bias, the study of Rimaz et al. was judged as ‘some concerns’ due to the lack of protocol registration.

### 3.4. Primary Outcomes: Pain Score

**Figure 3:**
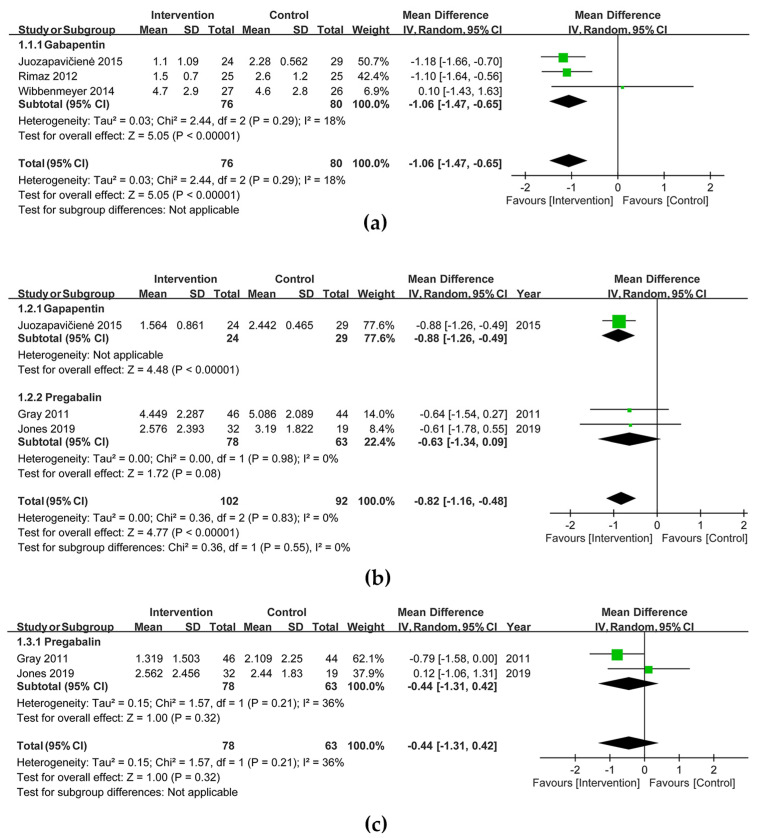
Forest plots of visual analogue scale (**a**) within 24 h, (**b**) from 72 h to 9 days and (**c**) after 3 weeks [17,18,19,20,21].

**Figure A1:**
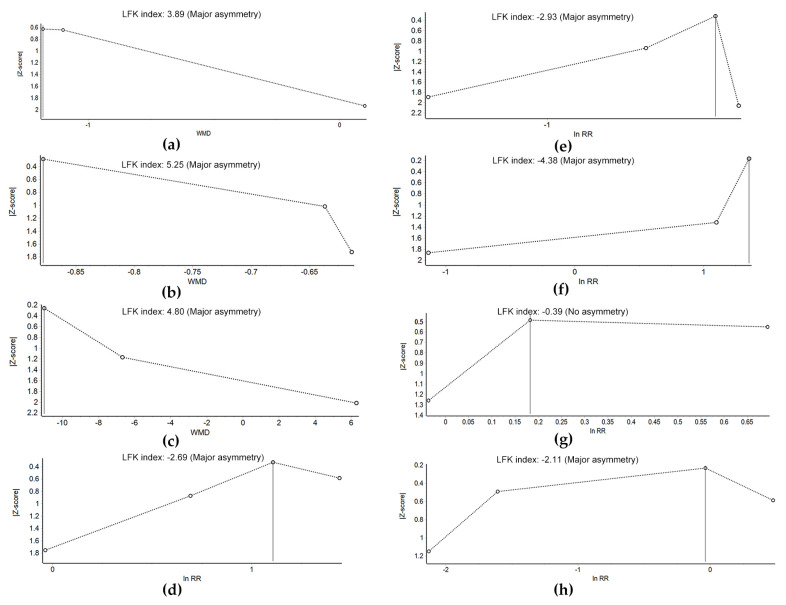
Doi plots of (**a**) visual analogue scale (VAS) 24 h, (**b**) VAS 72 h—9D, (**c**) opioid consumption 72 h–9D, (**d**) dizziness, (**e**) nausea, (**f**) drowsiness, (**g**) urinary retention and (**h**) pruritus. Abbreviations: LFK = Luis Furuya-Kanamori; ln RR = log scale relative risks; WMD = weighted mean difference.

**Figure A2:**
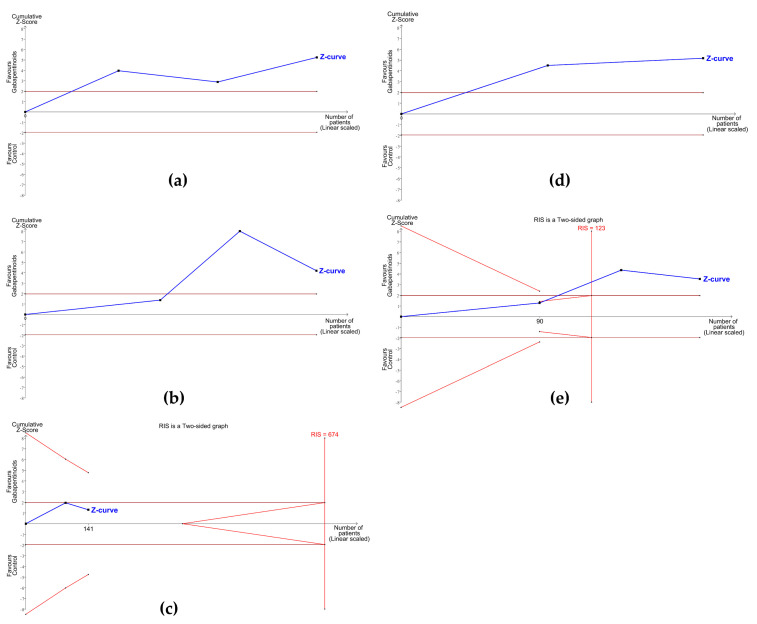
Trial sequential analysis (TSA) of visual analogue scale (**a**–**c**) (**a**) within 24 h, (**b**) 72 h to 9 days and (**c**) after 3 weeks. TSA of opioid consumption (**d**) within 24 h and (**e**) 72 h to 9 days. Abbreviation: RIS = required information size.

In the first 24 h after an acute burn injury, the analgesic effect showed significant difference in gabapentinoid-treated patients [MD (95% CI) = −1.06 (−1.47, −0.65), I^2^ = 18%; three trials; 156 participants] (Figure 3a). Pain score reduction was observed from 72 h to 9 days [MD (95% CI) = −0.82 (−1.16, −0.48), I^2^ = 0%; three trials; 194 participants] (Figure 3b). Three weeks after the burn injury, the pain level did not significantly decrease [MD (95% CI) = −0.44 (−1.31, 0.42), I^2^ = 36%; two trials; 141 participants] in gabapentinoid-treated patients (Figure 3c). The Doi plot yielded a major asymmetry with an LFK index of 3.89 and 5.25 for pain reduction within 24 h and from 72 h to 9 days, respectively (Figure A1a,b). Subgroup analysis between gabapentin and pregabalin in the group of 72 h to 9 days showed no significant difference (*p* = 0.55, I^2^ = 0%). TSA depicted that the O’Brien–Fleming monitoring boundaries and line of RIS were not renderable in the endpoints of pain score reduction in first 24 h (Figure A2a) and 72 h to 9 days (Figure A2b) because the end of the Z curve far exceeded the line of RIS. Both figures indicated true positive results. The end of the Z curve in the endpoints of pain score reduction 3 weeks later neither crossed the O’Brien–Fleming monitoring boundaries nor crossed futility boundaries, indicating a false-negative result (Figure A2c).

### 3.5. Primary Outcomes: Opioid Consumption

**Figure 4:**
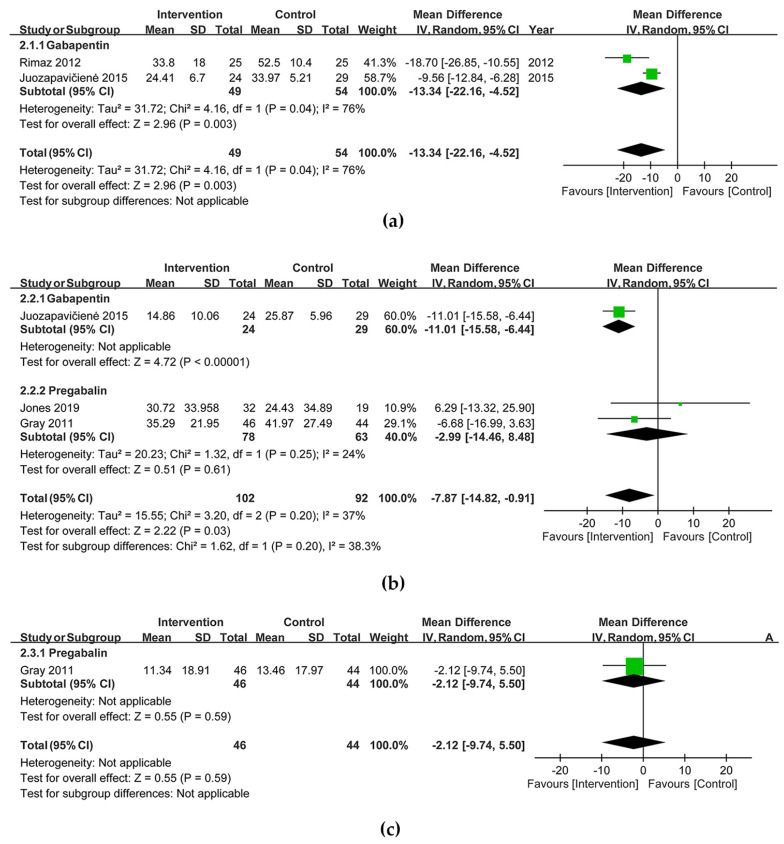
Forest plots of opioid consumption (**a**) within 24 h, (**b**) from 72 h to 9 days and (**c**) and 3 weeks [17,18,19,20].

Opioid consumption was reduced with the use of gabapentinoids within 24 h [MD (95% CI) = −13.34 mg ME (−22.16, −4.52), I^2^ = 76%; two trials; 103 participants] and from 72 h to 9 days [MD (95% CI) = −7.87 mg ME (−14.82, −0.91), I^2^ = 37%; three trials; 194 participants] (Figure 4a,b). Three weeks after the burn injury, no significant opioid-sparing effect was observed in gabapentinoid-treated patients [MD (95% CI) = −2.12 mg ME (−9.74, 5.50); one trial; 90 participants] (Figure 4c). The Doi plot yielded a major asymmetry with an LFK index of 4.8 for pain reduction from 72 h to 9 days (Figure A1c). The subgroup analysis in the group of 72 h to 9 days showed no significant difference (*p* = 0.20, I^2^ = 37%). The end of the Z curve far exceeded the line of RIS in the endpoint of opioid consumption within 24 h, so the O’Brien–Fleming monitoring boundaries and line of RIS were not renderable, indicating a true-positive result (Figure A2d). The end of the Z curve in the endpoint of opioid consumption between 72 h to 9 days not only crossed the O’Brien–Fleming monitoring boundaries but also the line of RIS (Figure A2e), indicating a positive result.

### 3.6. Secondary Outcomes: Adverse Events

Table 2 presents the adverse events reported. Dizziness [RR = 3.034 (95% CI = 1.006, 9.147, I^2^ = 0%)] and drowsiness [RR = 3.255 (95% CI = 1.135, 9.335, I^2^ = 0%)] were significantly more common in gabapentinoid-treated patients when using the IVhet model. The risks of nausea, diarrhoea, constipation, urinary retention and pruritus were not statistically increased compared with the control group. However, sensitivity analysis using the Bayesian approach for zero events did not support higher risks of dizziness, drowsiness or other adverse events in the gabapentin-treated group, except for a lower incidence of diarrhoea (Table 2). The Doi plots yielded a major asymmetry in most adverse outcomes, except for urinary retention, with an LFK index of −0.39 (Figure A1d–h).

### 3.7. CoE by GRADE Methodology

The GRADE assessment demonstrated very low, low and very low CoE in the outcomes of pain score reduction within 24 h, from 72 h to 9 days and after 3 weeks, respectively (Table 3). The CoE regarding opioid consumption within 24 h, from 72 h to 9 days and after three weeks was judged to be very low, very low and low, respectively (Table 3). The first domain of GRADE was downgraded by one to two levels due to the high proportion of studies with some concerns and/or high overall RoB. We also downgraded the domain of imprecision in some endpoints if an insufficient sample size and inconclusive result were detected by TSA or wide 95% CIs were reported. Publication bias was concerning because major asymmetries were observed in the Doi plots of most outcomes. Various adverse events were also classified as very low CoE due to the above-mentioned limitations (Table 4). 

## 4. Discussion

The adoption of gabapentinoids as a part of multimodal analgesia treatment for burn injuries has been recommended in several review articles and practice guidelines [2,22]. According to the American Burn Association Guideline, experts recommend considering the use of agents such as gabapentin or pregabalin as adjuncts to opioids for the treatment of neuropathic pain in patients who experience neuropathic pain or do not respond adequately to standard therapy (Level C). This recommendation was informed by several reports, including case series and case–control studies, as well as two RCTs that were specifically included in our meta-analysis [17,21]. Unfortunately, they did not employ the updated and comprehensive methodological approach to assess the certainty of the evidence. To the best of our knowledge, this meta-analysis provides the first comprehensive investigation into the effects of gabapentinoids on burn pain. Consequently, our study holds significant value as a reference for the future development of clinical guidelines in this domain. In our systematic review with a meta-analysis, gabapentinoids exhibited pain reduction and opioid-sparing effects within 24 h and from 72 h to 9 days. The effectiveness in subjective pain alleviation and morphine reduction did not last for 3 weeks but was still inconclusive in TSA. Gabapentinoids are not related to clinically significant adverse effects after sensitivity analysis using the Bayesian approach. Through comprehensive methodologies in evidence-based medicine, the results provide objective information for the use of gabapentinoids for burn patients.

The role of gabapentinoids in treating burn pain is still evolving. Cuignet et al. and Gray et al. reported early experiences and positive results of gabapentin in patients with an acute burn injury [22,23]. The latest guidelines for acute burn pain from the American Burn Association in 2020 suggest the adjunctive use of gabapentinoids for refractory burn pain and neuropathic pain, based on only two RCTs and three non-RCTs [2]. To provide updated and evidence-based recommendations, we included five RCTs in our systematic review and excluded studies if burn pain was elicited by experimental models, such as thermodes or intradermal capsaicin [24,25]. All of the included trials on the adult population applied baseline analgesics using morphine with or without other adjunctive medication. This implied that our analysis predominantly examined gabapentinoids as a component of a multimodal analgesic regimen, rather than as a standalone treatment for neuropathic pain. Pregabalin and gabapentin share a similar mechanism of action and have similar pharmacokinetics/pharmacodynamics; as such, we considered RCTs of both drugs for burn pain [5]. The transition between the two gabapentinoids could be achieved theoretically using pharmacokinetic models, which may facilitate clinical applications and delineate dose–response relationships [26]. However, their role in burn injury should be established due to the heterogenous endpoints in each RCT. Gray et al. and Jones et al. investigated the role of pregabalin in acute burn injures, but the former study failed to report adverse events [17,18]. Rimaz et al. provided detailed haemodynamic parameters, pain score and morphine consumption within the first 24 h after burn wound procedures [19]. Wibbenmeyer et al. and Juozapavičienė et al. analysed pain scores and adverse events of gabapentin up to 72 h [20,21]. Flexible dosage or a titration schedule was utilised in three trials, and a fixed dosage was administered in two studies [17,18,19,20,21]. The studies that used gabapentin reported pain scores and opioid consumption within days, while those that used pregabalin reported more long-term outcomes. The abovementioned conditions limited the strength of the pooled estimates from the sparse studies. TSA and the Bayesian approach were chosen to avoid the probability of false positive or false negative estimates and provide more objective results in terms of the effects of gabapentinoids on burn pain.

Burn pain consists of background, breakthrough and procedural pain due to the necessity of frequent wound management and surgical debridement [2]. The specific phases of acute burn pain and the precise timing of the transition from acute to chronic burn pain are still uncertain [1,27]. Therefore, it is challenging to establish an arbitrary and universally accepted grouping. To minimize heterogeneity, we divided the outcomes into three groups based on a review of the literature and the design of individual studies. In the first prospective quantitative study by Leazer et al., they observed a decrease in daily average opioid consumption and pain scores on the ninth day post-burn. Considering this finding, it is reasonable to evaluate the effects of gabapentinoids within the first nine days [28]. Monitoring boundaries or futility boundaries calculated with TSA have been widely applied to provide information regarding the precision and uncertainty of the meta-analysis results and to avoid spurious results from too few studies and participants [13]. Doi et al. introduced the IVhet model as a superior alternative to the traditional random-effects model for the meta-analysis of heterogeneous studies [29]. Jia et al. demonstrated that meta-analyses with rare or zero events were often underpowered and recommended post hoc analysis [30]. Methods such as continuity correction of single-zero-event studies and data exclusion of double-zero-event trials may result in misinterpretation [31]. We assessed the CoE in the domain of imprecision more carefully based on the results of TSA [32]. We used Bayesian analysis to examine studies with no events in one or both treatment arms, according to the framework proposed by Xu et al. [11]. We utilised Doi plots and LFK indexes instead of the conventional funnel plot with Egger’s regression for better detection and visualisation of publication bias [10]. Although low to very low CoE was judged in all endpoints, the pooled estimates indicate the possibility of potential benefits of gabapentinoids in reducing pain scores and opioid consumption in the acute phase of burn injuries. Although drawing a conclusion is difficult, adverse effects such as dizziness and drowsiness should be carefully monitored.

The efficacy of gabapentinoids in managing pain intensity and reducing opioid consumption following burn injury shows slight variations compared to previously published meta-analyses focusing on the perioperative utilization of gabapentinoids [33,34]. Fabritius et al. observed an opioid-sparing effect during the first 24 h with partial pain score reduction in pregabalin-treated adult surgical patients [33]. Regarding the perioperative use of gabapentinoids, Verret et al. reported a slight reduction in pain and a decrease in cumulative opioid dosage within the initial 72 h [34]. However, these effects did not reach a clinically significant level of analgesic efficacy. This discrepancy can be attributed to the differences in underlying pathophysiology between burn injuries and postoperative pain. The analgesic benefit of gabapentinoids in acute burn injury may be influenced by several factors. First, patients with major burn injuries often undergo resuscitative and hypermetabolic phases, leading to physiologic fluid shifts among body compartments and highly variable serum protein concentrations [35]. The volume of distribution for gabapentin and pregabalin resembles that of total body water due to their high aqueous solubility and lack of significant tissue- or protein-binding ability [5]. The circulatory derangement may pose great challenges in pharmacokinetic and pharmacodynamic monitoring; thus, effective dosages should be tailored according to clinical scenarios. Secondly, second-degree burn injuries are notorious for their severe pain, and the time required for complete re-epithelialisation varies among individuals. Leazer et al. reported a significant positive correlation of opioid consumption with the total body surface area and a negative correlation with patient’s age [28]. Lastly, the pathophysiology of burn pain involves peripheral and central processes with combined features of acute nociceptive, inflammatory and neuropathic pain in different time periods [1]. The effectiveness of gabapentinoids may be attributed to the reduction in neuropathic pain and modulation of hyperalgesia after thermal insults rather than pain caused by other mechanisms.

Gabapentinoids exhibit adverse effects on the central nervous, respiratory and gastrointestinal systems [36]. Adverse events are common and frequently elicit discontinuation of the medication [37]. Coadministration of gabapentinoids and opioids are thought to be associated with respiratory or cognitive depression in patients with risk factors such as old age or chronic kidney disease [38,39]. In our meta-analysis, adverse effects were reported in four trials, and no respiratory distress was described along with opioid use. The risks of dizziness and drowsiness increased when using the IVhet model but were not different when using the Bayesian approach, indicating uncertain evidence. The incidence rates of other adverse events such as nausea, constipation, urinary retention and pruritus were not elevated. The safety of gabapentin in acute pain management is still controversial. Fabritus et al. reported no significant difference in adverse events related to the use of gabapentin in postoperative pain management [40]. By contrast, Verret et al. found elevated risks of dizziness and visual disturbance during perioperative use of gabapentinoids, with a lower risk of postoperative nausea and vomiting [34]. Most adverse reactions involving the central nervous system have a clear dose–response relationship, but the pharmacokinetic and pharmacodynamic monitoring in acute burn injury may be arduous [36]. The optimal dose or frequency of administration of gabapentin and pregabalin remains to be elucidated. Therefore, careful patient selection and timely adjustment according to clinical responses are mandatory.

Our study has several limitations. Firstly, the number of included studies and participants were limited. Only five trials were enrolled in our meta-analysis. Secondly, the existence of different regimens and titration algorithms among studies was inevitable, and the cumulative dose of each participant was not reported. Furthermore, the absence of alternative pain assessment tools in situations where the VAS was not feasible and the lack of specified burn surface area in the included studies could potentially impact the clinical applicability of the findings. To explore the dose-dependent relationship of gabapentinoids through meta-regression, further results from larger clinical studies are required in the future for validation.

## 5. Conclusions

In this systematic review with meta-analysis and TSA, gabapentinoids were found to provide modest analgesic benefits to burn patients during the first three weeks following the injury. A trend of increased risk of drowsiness and dizziness was observed, so the adverse effects during prescription should be carefully monitored. More large-scale RCTs are still warranted for the reinforcement of CoE in clinical use.

## Figures and Tables

**Table 1:** Characteristics of included studies.

Participants Characteristics						Interventions		Comparison	Outcomes	
Study	Year	Country	Funding	Study Design	Baseline Analgesics	Experimental Groups (n)	Administration	Control Group (n)	Outcome Analysed	Study Follow-Up
Gray et al. [17]	2011	Australia	Pfizer Australia and Royal Brisbane and Women’s Hospital Foundation	Single-centre, double-blind RCT (ISRCTN56448626)	Acetaminophen, opioid (oral, PCA)	Pregabalin (34)	75 mg–300 mg BID, PO, 28 days *	Placebo (33)	NPS (0–10) Opioid consumption	Weekly, up to 4 weeks
Rimaz et al. [19]	2012	Iran	None declared	Double-blind, placebo-controlled RCT	IV morphine (PCA)	Gabapentin (25)	1200 mg, single dosing, PO	Placebo (25)	VAS (0–100) Opioid consumption Adverse effects	24 h
Wibbenmeyer et al. [21]	2014	USA	None declared	Single-centre, double-blind, placebo-controlled RCT (NCT01265056)	Acetaminophen, NSAIDs and morphine	Gabapentin (27)	1200 mg once, followed by 300–1200 mg TID, PO *	Placebo (26)	NRS (0–10) Adverse effects	Pain: day 3 or discharge Opioids: until first clinic visit
Juozapavičienė et al. [20]	2015	Lithuania	None declared	Single-centre, parallel-designed RCT	IV morphine (PCA)	Gabapentin (24)	1200 mg/day PO	Control (29)	VAS (0–100) Opioid consumption Adverse effects	Up to 72 h
Jones et al. [18]	2019	USA	Pfizer Inc.	Single-centre, double-blind, placebo-controlled RCT	IV and oral opioids	Pregabalin (32)	150 mg BID PO * 300 mg BID PO *	Placebo (19)	VAS (0–100) Opioid consumption Adverse effects	Days 9, 25, 90 and 180

* Administration with titration and tapering protocol. The dosage range indicated adjustment according to clinical requirement. Abbreviations: BID = twice daily; IV = intravenous; NPS = numeric pain scale; NRS = numeric rating scale; PCA = patient-controlled analgesia; PO = orally; RCT = randomised controlled trial; TID = three times a day; VAS = visual analogue scale.

**Table 2:** Adverse events after gabapentinoid administration for burn pain.

Adverse Events	Gabapentin			Pregabalin		Pooled		LFK Index
	No. of Trials	RR (95% CI) IVhet Model	Bayesian Analysis (95% CrI)	No. of Trials	RR (95% CI) IVhet Model	RR (95% CI) IVhet Model	Bayesian Analysis (95% CrI)	
Dizziness	3	3.035 (0.925, 9.952)	1.62 (0.338, 6.28)	1	3.030 (0.153, 59.967)	3.034 (1.006, 9.147, I^2^ = 0%)	1.58 (0.413, 5.25)	−2.69
Nausea	3	0.791 (0.358, 1.750)	0.628 (0.256, 1.43)	1	0.202 (0.009, 4.724)	0.729 (0.338, 1.575, I^2^ = 0%)	0.419 (0.075, 1.33)	−2.93
Drowsiness	3	3.255 (1.135, 9.335)	1.69 (0.285, 6.06)	0	N/A	3.255 (1.135, 9.335, I^2^ = 0%)	1.69 (0.285, 6.06)	−4.38
Diarrhoea	2	0.359 (0.034, 3.831)	1.01 × 10^−0.7^ (8.79 × 10^−21^, 0.125)	0	N/A	0.359 (0.034, 3.831, I^2^ = 0%)	1.01 × 10^−0.7^ (8.79 × 10^−21^, 0.125)	N/A
Constipation	2	0.993 (0.183, 5.394)	0.806 (0.340, 2.20)	0	N/A	0.993 (0.183, 5.394, I^2^ = 0%)	0.806 (0.340, 2.20)	N/A
Urinary retention	3	1.542 (0.260, 9.135)	0.475 (0.0633, 2.67)	0	N/A	1.542 (0.260, 9.135, I^2^ = 0%)	0.475 (0.0633, 2.67)	−0.39
Pruritus	3	1.094 (0.329, 3.641)	0.337 (0.0343, 2.06)	1	0.119 (0.015, 0.942)	0.625 (0.134, 2.908, I^2^ = 38%)	0.444 (0.0643, 1.98)	−2.11

Abbreviations: CI = confidence interval; CrI = credible interval; IVhet = inverse variance heterogeneity; LFK = Luis Furuya-Kanamori; N/A = not applicable; RR = risk ratio.

**Table 3:** GRADE assessment: gabapentinoids compared to placebo for post-burn pain: primary outcomes.

Certainty Assessment							No. of Patients		Effect	Certainty
No. of Studies	Study Design	Risk of Bias	Inconsistency	Indirectness	Imprecision	Other Considerations	Gabapentinoids	Control	Absolute (95% CI)	
Pain score reduction 24 h										
3	Randomised trials	Very serious ^a^	Not serious	Not serious	Not serious	Publication bias strongly suspected ^b^	76	80	MD 1.06 lower (1.47 lower to 0.65 lower)	⨁◯◯◯ Very low
Pain score reduction 72 h to 9 days										
3	Randomised trials	Serious ^c^	Not serious	Not serious	Not serious	Publication bias strongly suspected ^b^	102	92	MD 0.82 lower (1.16 lower to 0.48 lower)	⨁⨁◯◯ Low
Pain score reduction 3 weeks										
2	Randomised trials	Serious ^d^	Not serious	Not serious	Very serious ^e^	Publication bias strongly suspected ^b^	78	63	MD 0.44 lower (1.31 lower to 0.42 higher)	⨁◯◯◯ Very low
Opioid consumption 24 h										
2	Randomised trials	Very serious ^a^	Not serious	Not serious	Not serious	Publication bias strongly suspected ^b^	49	54	MD 13.34 mg/day lower (22.16 lower to 4.52 lower)	⨁◯◯◯ Very low
Opioid consumption 72 h to 9 days										
3	Randomised trials	Very serious ^f^	Not serious	Not serious	Not serious	Publication bias strongly suspected ^b^	102	92	MD 7.87 mg/day lower (14.82 lower to 0.91 lower)	⨁◯◯◯ Very low
Opioid consumption 3 weeks										
1	Randomised trials	Serious ^d^	Not serious	Not serious	Serious ^g^	None	46	44	MD 2.12 mg/day fewer (9.74 fewer to 5.5 more)	⨁⨁◯◯ Low

Abbreviations: CI = confidence interval; MD = mean difference; RR = risk ratio. ^a^ More than half of enrolled randomised controlled trials (RCTs) were judged as high overall risk of bias. ^b^ Major asymmetry from Doi plot. ^c^ More than quarter of enrolled RCTs were judged as high overall risk of bias. ^d^ More than half of enrolled RCTs were judged as some-concern overall risk of bias. ^e^ Inconclusive result and insufficient sample size calculated by trial sequential analysis, and wide 95% confidence interval (CI) of pooled estimates. ^f^ One RCT was judged as high overall risk of bias and two RCTs were judged as some-concern overall risk of bias. ^g^ Wide 95% CI.

**Table 4:** GRADE assessment: gabapentinoids compared to placebo for post-burn pain: adverse events.

Certainty Assessment							No. of Patients		Effect		Certainty
No. of Studies	Study Design	Risk of Bias	Inconsistency	Indirectness	Imprecision	Other Considerations	Gabapentinoids	Control	Relative (95% CrI)	Absolute (95% CrI)	
Dizziness											
4	Randomised trials	Very serious ^a^	Not serious	Not serious	Very serious ^b^	Publication bias strongly suspected ^c^	11/108 (10.2%)	3/99 (3.0%)	RR 1.58 (0.41 to 5.25)	18 more per 1000 (from 18 fewer to 129 more)	⨁◯◯◯ Very low
Nausea											
4	Randomised trials	Very serious ^a^	Not serious	Not serious	Serious ^d^	Publication bias strongly suspected ^c^	8/108 (7.4%)	12/99 (12.1%)	RR 0.419 (0.075 to 1.330)	70 fewer per 1000 (from 112 fewer to 40 more)	⨁◯◯◯ Very low
Drowsiness											
3	Randomised trials	Very serious ^a^	Not serious	Not serious	Very serious ^b^	Publication bias strongly suspected ^c^	17/76 (22.4%)	6/80 (7.5%)	RR 1.690 (0.285 to 6.060)	52 more per 1000 (from 54 fewer to 379 more)	⨁◯◯◯ Very low
Diarrhoea											
2	Randomised trials	Very serious ^a^	Not serious	Not serious	Serious ^d^	None	0/52 (0.0%)	2/51 (3.9%)	RR 0.000 (0.000 to 0.125)	-- per 1000 (from 34 fewer to --)	⨁◯◯◯ Very low
Constipation											
2	Randomised trials	Very serious ^a^	Not serious	Not serious	Serious ^d^	None	2/52 (3.8%)	2/51 (3.9%)	RR 0.806 (0.340 to 2.200)	8 fewer per 1000 (from 26 fewer to 47 more)	⨁◯◯◯ Very low
Urinary retention											
3	Randomised trials	Very serious ^a^	Not serious	Not serious	Very serious ^b^	None	2/76 (2.6%)	1/80 (1.3%)	RR 0.4750 (0.0633 to 2.6700)	7 fewer per 1000 (from 12 fewer to 21 more)	⨁◯◯◯ Very low
Pruritus											
4	Randomised trials	Very serious ^a^	Not serious	Not serious	Serious ^d^	Publication bias strongly suspected ^c^	5/108 (4.6%)	5/99 (5.1%)	RR 0.4440 (0.0643 to 1.9800)	28 fewer per 1000 (from 47 fewer to 49 more)	⨁◯◯◯ Very low

Abbreviations: CrI = credible interval; RR = risk ratio. ^a^ More than half of enrolled randomised controlled trials (RCTs) were judged as high overall risk of bias. ^b^ Very wide 95% credible interval (CrI). ^c^ Major asymmetry from Doi plot. ^d^ Wide 95% CrI.

## Data Availability

Not applicable.

## Supplementary Materials

The following supporting information can be downloaded at: https://www.mdpi.com/article/10.3390/jcm12155042/s1, Table S1: Search strategy for individual databases; Table S2: PRISMA 2020 checklist. Reference [8] is cited in Supplementary Materials.
