# Supplementary material for: Effectiveness and Adverse Events of Gabapentinoids as Analgesics for Patients with Burn Injuries: A Systematic Review with Meta-Analysis and Trial Sequential Analysis
Source: J Clin Med. 2023 Jul 31;12(15):5042. doi: 10.3390/jcm12155042 (PMC10420087; doi:10.3390/jcm12155042)
Supplement: Supplementary file 1 [file jcm-12-05042-s001.zip › Supp. Table S1.pdf]

Supplementary Table S1. Search strategy for individual databases

Pubmed

1.	(burn[MeSH Terms]) AND ((pain[MeSH Terms]) OR (analgesic agents[MeSH Terms]))	1,639
2.	burn pain[Title/Abstract] OR post-burn[Title/Abstract] OR burn injury[Title/Abstract] OR (burn AND (neuropathic pain[Title/Abstract] OR neuralgia[Title/Abstract] OR neuropathy[Title/Abstract] OR analgesics[Title/Abstract] OR analgesia[Title/Abstract]))	9,982
3.	#1 OR #2	10,985
4.	gaba*[Title/Abstract] OR enacarbil[Title/Abstract] OR imagabalin[Title/Abstract] OR mirogabalin [Title/Abstract] OR pregabalin[Title/Abstract]	87,049
5.	(Gabapentin[MeSH Terms]) OR (Pregabalin[MeSH Terms])	6,036
6.	#4 OR #5	87,863
7.	#3 AND #6	56

Embase

1.	'burn'/exp OR 'burn'	120,578
2.	'pain'/exp OR 'pain'	1,935,221
3.	'analgesics'/exp OR 'analgesics'	1,099,092
4.	#1 AND (#2 OR #3)	14,949
5.	'burn pain':ti,ab OR 'post-burn':ti,ab OR 'burn injury':ti,ab OR (burn AND ('neuropathic pain':ti,ab OR 'neuralgia':ti,ab OR 'neuropathy*':ti,ab OR 'analgesics':ti,ab OR 'analgesia':ti,ab))	13,677
6.	#4 OR #5	25,653
7.	'gabapentin'/exp OR 'gabapentin'	36,114
8.	'pregabalin'/exp OR 'pregabalin'	17,502
9.	gaba*:ti,ab OR enacarbil:ti,ab OR imagablin:ti,ab OR mirogabalin:ti,ab	105,917
10.	#7 OR #8 OR #9	138,381
11.	#6 AND #10	458

CENTRAL

1.	MeSH descriptor: [burns] explode all trees	1,883
2.	MeSH descriptor: [pain] explode all trees	55,805
3.	MeSH descriptor: [analgesics] explode all trees	22,565
4.	#1 AND (#2 OR #3)	238

5.	'burn pain':ti,ab OR ‘post-burn’:ti,ab OR 'burn injury':ti,ab OR (burn AND ('neuropathic pain':ti,ab OR 'neuralgia':ti,ab OR 'neuropathy*':ti,ab OR ‘analgesics':ti,ab OR ‘analgesia’:ti,ab))	2,340
6.	#4 OR #5	2,383
7.	MeSH descriptor: [Gabapentin] explode all trees	912
8.	MeSH descriptor: [Pregabalin] explode all trees	901
9.	(gaba* OR *gabalin OR enacarbil OR imagablin OR mirogabalin):ti,ab	6,268
10.	#7 OR #8 OR #9	6,373
11.	#6 AND #10	36

SCOPUS

1.	TITLE-ABS-KEY(‘burn’ OR 'burn pain' OR 'post-burn' OR 'burn injury')	48,027
2.	TITLE-ABS-KEY('neuropathic pain' OR 'neuralgia' OR 'neuropathy' OR 'analgesics' OR 'analgesia')	48,389
3.	#1 AND #2	193
4.	TITLE-ABS-KEY(gabapentin OR pregabalin)	39,520
5.	TITLE-ABS-KEY(gaba* OR enacarbil OR imagablin OR mirogabalin)	137,407
6.	#4 OR #5	144,296
7.	#3 AND #6	56

EBSCO/CINAHL

1.	AB (‘burn’ OR 'burn pain' OR 'post-burn' OR 'burn injury') AND AB (analgesics)	10,009
2.	AB (pregabalin or lyrica) OR AB (gabapentin or neurontin) OR AB (gaba* OR *gabalin OR enacarbil OR imagablin OR mirogabalin)	2,074
3.	#1 AND #2	268

Limiter: human study

CNKI

1.	(AB='普瑞巴林' or AB='加巴噴丁'or AB='恩那卡比')	1,077
2.	(SU='疼痛') and ((FT='燒傷') or (FT='燒燙傷'))	6,788
3.	#1 AND #2	15
